# Exosomes Derived From Mesenchymal Stem Cells Ameliorate Renal Ischemic-Reperfusion Injury Through Inhibiting Inflammation and Cell Apoptosis

**DOI:** 10.3389/fmed.2019.00269

**Published:** 2019-11-19

**Authors:** Long Li, Rulin Wang, Yichen Jia, Ruiming Rong, Ming Xu, Tongyu Zhu

**Affiliations:** ^1^Department of Urology, Zhongshan Hospital, Fudan University, Shanghai, China; ^2^Shanghai Key Laboratory of Organ Transplantation, Shanghai, China; ^3^Department of Urology, The First Affiliated Hospital of Zhengzhou University, Zhengzhou, China

**Keywords:** mesenchymal stem cells, exosomes, ischemic-reperfusion injury, inflammation, apoptosis

## Abstract

This study aimed to investigate the underlying mechanism of mesenchymal stem cells (MSCs) on protection of renal ischemia reperfusion injury (IRI). Exosomes originated from MSCs (MSC-ex) were extracted according to the instructions of Total Exosome Isolation Reagent. Rats were divided into five groups: sham-operated, IRI, MSC, MSC-ex, and MSC-ex + RNAase group. MSCs or MSC-ex were injected via carotid artery. The renal function test and pathological detection were applied to determine the renoprotection of MSC-ex on IRI. Western blotting and quantitative reverse transcription polymerase chain reaction (RT-qPCR) were conducted to examine the levels of apoptosis-related proteins and inflammatory cytokines. Our results revealed that MSC-derived exosomes attenuated renal dysfunction, histologic damage, and decreased apoptosis. The expression levels of inflammatory cytokines, such as interleukin 6 (IL-6), tumor necrosis factor-α (TNF-α), nuclear factor kappa B (NF-κB), and interferon gamma (IFN-γ), were decreased by the MSC-ex treatment. The expression levels of caspase-9, cleaved caspase-3, Bax, and Bcl-2 caused by IR were also inhibited by MSC-ex. MSC-ex + RNAase group shared the similar pattern of changes with IRI group, likely due to the ability of RNA hydrolase to eliminate the function of exosomes. Our results demonstrated that exosomes originating from MSCs have protective effects on IRI via inhibiting cell apoptosis and inflammatory responses. Out findings may provide a new insight into therapeutic mechanism of MSCs on renal IRI.

## Introduction

Ischemia reperfusion injury (IRI), one of the major causes of post-operative renal allograft complications, is inevitable during transplantation (1, 2). It has been reported that IRI may cause acute renal failure and delayed graft function, thereby impairing long-term survival of the graft and recovery in post-transplantation (3–5). Therefore, understanding the pathogenesis may assist scholars to present new strategies to prevent IRI after renal transplantation.

Mesenchymal stem cells (MSCs) have been found to exert several biological functions, such as repairing tissue damage, suppressing inflammatory responses, and modulating the immune system (6, 7). They may protect acute kidney injury (AKI) induced by cisplatin, glycerol, and IRI in rats, however, the underlying mechanism has still remained elusive. Accumulating evidence supported that MSCs act in a paracrine manner (8). Therefore, the biological factors in conditioned medium, including exosomes and soluble factors, derived from MSCs have been extensively studied in recent years. Exosomes were reported to activate signaling pathways by binding to receptors (9–11). Compared with MSCs, exosomes are more stable and reservable, have no risk of aneuploidy, with lower possibility of immune rejection following allogeneic administration, and may provide alternative therapies for a variety of diseases (12). A previous study demonstrated that exosomes from MSC can reduce remnant kidney fibrosis in a 5/6 subtotal nephrectomy mice model (13). Furthermore, it has been revealed that infusing exosomes from MSC reduce the expression levels of 8- hydroxy-2′ -deoxyguanosine (8-OHdG), malonaldehyde (MDA), Bax, and caspase-3 in a cis-platinum-induced AKI mouse model (14). However, to date, the underlying mechanism of MSC-derived exosomes on renoprotection of IRI has remained obscure. Thus, the present study aimed to identify the protective effects and mechanism of MSC-ex on renal IRI.

Nuclear factor kappa B (NF-κB) is a family of dimeric transcription factors that participate in inflammatory responses, innate and adaptive immunity during kidney transplantation (15–17). It has been reported that MSCs can protect tissues against injury by regulating the activation of NF-κB. In the present research, we demonstrated that: (1) MSC-ex mitigated IRI-induced renal structural injury and improved renal function in rats; (2) the renoprotection of MSC-ex is due to downregulation of inflammatory factors and suppressing NF-κB signaling pathway.

## Materials and Methods

### Animals

Male Sprague-Dawley (SD) rats (weight, 200–250 g) were purchased from Shanghai SLAC Laboratory Animal Co., Ltd. (Shanghai, China), and housed in temperature-controlled, SPF condition with free access to food and water. Animals were fasted for 1 day prior to surgery. All animal procedures were performed in accordance with the guidelines of the bioethics, and the study was approved by the Ethics Committee of Zhongshan Hospital Affiliated to Fudan University (Shanghai, China).

### Separation, Cultivation, and Authentication of MSCs

The SD-MSC line RASMX-01001 was purchased from Cyagen Biosciences (Guangzhou, China). Cells were cultured at 37°C with 5% CO_2_ under humidity conditions.

### Extraction and Authentication of Exosomes

MSCs were cultivated overnight without serum according to the instructions of Total Exosome Isolation Reagent (Thermo Fisher Scientific, Waltham, MA, USA). Then, culture fluid was collected, and centrifuged at 2,000 g for 30 min at 4°C. The Total Exosome Isolation Reagent was added and mixed thoroughly, and the liquid was stored at 4°C overnight. The liquid was then centrifuged at 1,000 g for 60 min at 4°C, and supernatant was removed, and the exosomes were left at the bottom of the centrifuge tubes. Phosphate-buffered saline (PBS) was used to re-suspend exosomes, which was diluted to 100 μg/ml, and stored at −80°C until further analysis. The isolated exosomes were identified by transmission electron microscopy (TEM) (Supplementary Image 1) and detected by flow cytometry (Supplementary Image 2).

### An *in vivo* Model of Renal IRI

SD rats were anesthetized by intraperitoneal injection of pentobarbital at 0.1 g/kg. All rats were divided into five groups: sham-operated group, IR group, IR + MSC group (IR + MSC, 2 ^*^10^6^ MSC/1 ml PBS), IR + MSC-ex group (exosome 100 μg/ml in PBS), and IR + MSC-ex + RNAase (exosome 100 μg/ml in PBS). Renal IRI was induced by clamping the left renal artery via a median abdominal incision for 45 min, plus a right nephrectomy. Following clamp removal, adequate restoration of blood flow was made before abdominal closure and the right kidney was removed. The left carotid artery was separated by para-tracheal incision using a 24G arteriovenous puncture needle to make the carotid puncture. Sham-operated animals underwent the same surgical procedure without clamping. The MSCs (1.5 × 10^5^ cells in PBS), exosomes (30 μg in PBS) or RNAase were given via carotid artery 1 h after I/R.

### Assessment of Renal Function

Whole blood was centrifuged at 1,600 g for 25 min at 4°C to obtain serum. An auto biochemistry instrument was used to measure the levels of serum creatinine (SCr) and blood urea nitrogen (BUN).

### Histological Assessment

Hematoxylin and eosin (H&E) staining was performed to assess histological injury. The tissue sections were blind-labeled and reviewed by two pathologists. Renal damage was graded based on the percentage of damaged tubules in the sample: 0 = normal kidney (no damage); 1 = minimal damage (<25% damage); 2 = mild damage (25%−50% damage); 3 = moderate damage (50%−75% damage); 4 = severe damage (75%-100% damage); and 5 = extremely damaged (100% damage), as previously described. Injury included inflammatory cell infiltration, dilation of renal tubules, and interstitial edema. Scores of 1 or 2 represent mild injury, and scores of 3 or 4 and 5 represent moderate and severe injuries, respectively. The scores are the average of two reads.

### Detection of Apoptosis

The terminal deoxynucleotidyl transferase-mediated dUTP-biotin nick end labeling (TUNEL) assay was used to detect apoptotic cells according to the manufacturer's instructions. Apoptotic cells were examined at 400× magnification over 20 fields of tubular areas.

### Quantitative Reverse Transcription Polymerase Chain Reaction (RT-qPCR)

Total RNA was extracted from rat kidneys by TRIzol reagent (Invitrogen, Carlsbad, CA, USA) according to the manufacturer's instructions. Total RNA (3–5 μg) was transcribed into cDNA by Superscript II reverse transcriptase and random primer oligonucleotides. Our gene-specific primers, such as interleukin 6 (IL-6), tumor necrosis factor-α (TNF-α), NF-κB, and interferon gamma (IFN-γ), are presented in Table 1.

**Table 1:** Sequences of gene-specific primers used in RT-qPCR.

IL-6	Forward	5′-CGAGCCCACCAGGAACGAAAGTC-3′
	Reverse	5′-CTGGCTGGAAGTCTCTTGCGGAG-3′
TNF-α	Forward	5′ -CCTTATCTACTCCCAGGTTCTC-3′
	Reverse	5′-AGGGGCCATCCACAGTCTTC-3′
NF-κB	Forward	5′-TGTCCATGCAGCTTCGGCGG-3′
	Reverse	5′-GGCCGGGGTTCAGTTGGTCC-3′
IFN-γ	Forward	5′-AAAGACAACCAGGCCATCAG-3′
	Reverse	5′-CTTTTCCGCTTCCTTAGGCT-3′

### Western Blot Analysis

Renal tissue homogenates were prepared, and the supernatant was maintained at 4°C. Besides, 30 μg protein from each sample was separated by sodium dodecyl sulfate-polyacrylamide gel electrophoresis (SDS-PAGE), and then transferred onto polyvinylidene fluoride (PVDF) membranes. The primary antibodies, including anti-cleaved caspase-3 (Cell Signaling Technology, Inc., Danvers, MA, USA), anti-NF-κB (Cell Signaling Technology, Inc., Danvers, MA, USA), anti-p-NF-κB (Cell Signaling Technology, Inc., Danvers, MA, USA), anti-IκB (Cell Signaling Technology, Inc., Danvers, MA, USA), and anti-p-IκB (Cell Signaling Technology, Inc., Danvers, MA, USA) were added and incubated at 4°C overnight, followed by incubation with horseradish peroxidase (HRP)-conjugated secondary antibodies at room temperature. Immunoreactive bands were visualized using ECL Western Blotting Substrate (Thermo Fisher Scientific, Waltham, MA, USA). For the loading control, the same membranes were simultaneously probed with anti-GAPDH (Abcam, Cambridge, UK). The signals were quantified by scanning densitometry using a Bio-Image Analysis System. Relative protein expression was subsequently quantitated by normalizing to GAPDH levels using the Image-Pro plus 6.0 software.

### Statistical Analysis

Data were expressed as mean ± standard deviation (SD), and statistical analysis was performed by Student's *t*-test or one-way analysis of variance (ANOVA) with *post-hoc* Student-Newman-Keuls test. *P* < 0.05 was considered statistically significant.

## Results

### Exosomes Attenuated Renal Dysfunction, Histologic Damage, and Decreased Apoptosis

**Figure 1:**
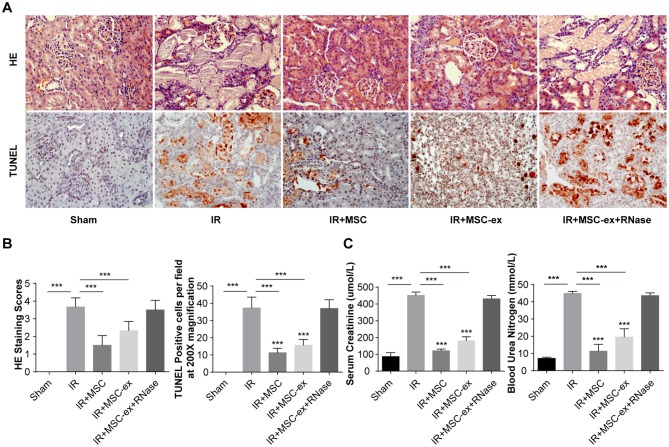
MSCs and exosomes ameliorated renal IRI. **(A)** Representative images of HE and TUNEL staining methods in rat kidneys with administration of MSC, MSC-ex, and MSC-ex+RNAase after IR. Images were captured at x 200× magnification, scale bar = 10 μm. The levels of SCr and BUN were measured using the peripheral blood automatic biochemical analyzer. **(B)** Shows scores of HE staining and the number of TUNEL positive cells. Histopathological grading of tissue injury was assessed using the 0- to 4-point scoring system. **(C)** The levels of SCr and BUN were measured using the peripheral blood automatic biochemical analyzer. ^***^P < 0.001, *n* = 6.

To evaluate the renoprotective effects of exosomes from MSCs, we analyzed two indicators of renal function, including BUN and SCr. Rats underwent renal IRI showed significant increase in the levels of SCr (482 ± 37 vs. 86 ± 24 μmol/L) and BUN (45.42 ± 2.73 vs. 7.13 ± 0.74 mmol/L) compared with the sham-operated group. Additionally, MSC treatment and MSC-ex treatment decreased the levels of SCr (148 ± 16 μmol/L; 231 ± 30 μmol/L) and BUN (20.49 ± 5.70 mmol/L; 31.36 ± 5.53 mmol/L), while this protective effect was not observed in the MSC-ex + RNAase group (Figure 1C).

Moreover, H&E and TUNEL staining methods were performed to assess the degree of renal injury and tissue apoptosis in each treatment group. The 0- to 4-point scoring system was used to evaluate tissue injury. The results demonstrated that rats that underwent renal IRI had tissue injury, increased inflammatory cell infiltration, and larger areas of tubular necrosis, vacuolization and cast formation. Rats treated with MSC and MSC-ex experienced significant attenuation of pathological damage in kidney, while no significant therapeutic effect was found on rats in MSC-ex + RNAase group (Figures 1A,B).

TUNEL staining demonstrated that cell apoptosis was increased after IRI. In addition, treatment with MSC and MSC-ex remarkably decreased cell apoptosis, while no significant decrease was observed in the MSC-ex + RNAase group (Figures 1A,B).

### Exosomes Alleviated Inflammatory Responses by Reducing the Levels of TNF-α, NF-κB, IFN-γ, and IL-6

**Figure 2:**
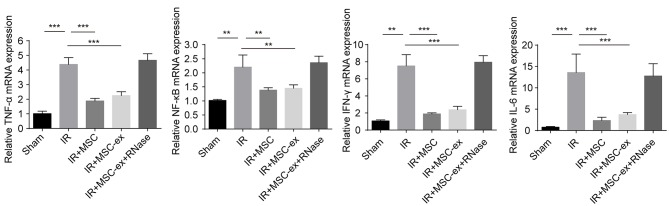
MSCs and exosomes from MSCs decreased proinflammatory Total RNA was extracted from renal tissue 48 h after IR, and then reversely transcribed into cDNA. Treatment with MSCs or MSC-ex reduced the mRNA levels of TNF-α, NF-κB, IFN-γ, and IL-6, while MSC-ex + RNAase had no influence, ^**^P < 0.01, ^***^P < 0.001, *n* = 6.

To detect the anti-inflammatory function of exosomes, we further assessed the mRNA level of inflammatory cytokines and transcription factors. Results showed that mRNA levels of TNF-α, NF-κB, IFN-γ, and IL-6 were significantly increased in IRI, while those levels were reduced after undergoing MSC and MSC-ex. The MSC-ex+RNAase had no significant effect. The result suggested that MSC-ex may have a protective role in IRI through suppressing inflammation (Figure 2).

### Exosomes Inhibited NF-κB Signaling Pathway and Inflammatory Response

**Figure 3:**
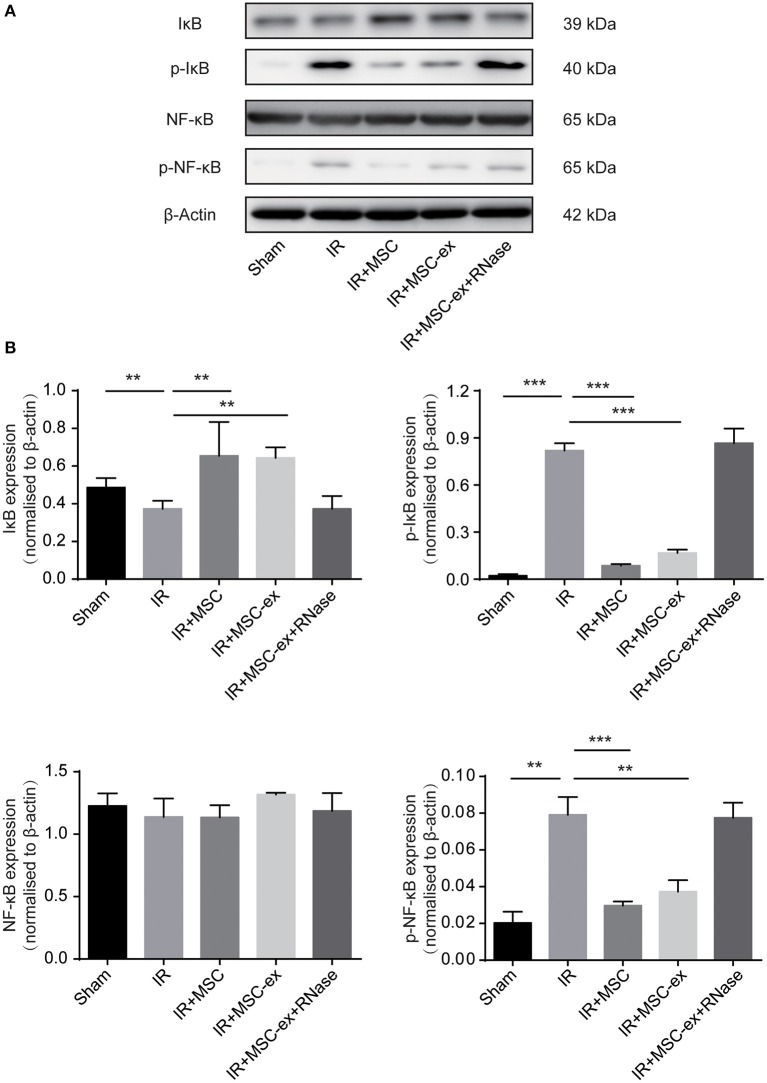
MSC and exosomes from MSC inhibited the NF-κB signaling pathway by decreasing expression levels of p-NF-κB and p-IκB, and increasing expression level of IκB. **(A)** Representative images show the protein levels of IκB, p-IκB, NF-κB, and p-NF-κB. **(B)** The expression levels of IκB, p-IκB, NF-κB, and p-NF-κB were normalized to β-actin levels within the same sample. ^**^P < 0.01, ^***^P < 0.001, *n* = 6.

It has been reported that activation and translocation of NF-κB can be involved in pro-inflammatory responses. Therefore, we detected the levels of phosphorylated NF-κB and IκB in renal tissue of rats in each group. The results revealed that protein levels of p-NF-κB and p-IκB were increased after renal IR. On the contrary, the level of IκB, which is an inhibitor of NF-κB, was decreased after IR, and the expression of NF-κB did not remarkably change. Both MSC and MSC-ex inhibited IR-induced increase of p-NF-κB, p-IκB and decrease of IκB. The expression levels of NF-κB, IκB, p-IκB, and p-NF-κB in IR + MSC-ex + RNAase group were similar to those of IRI (Figure 3).

### Exosomes Inhibited Activation of Caspase-3 and Cell Apoptosis Caused by IRI

**Figure 4:**
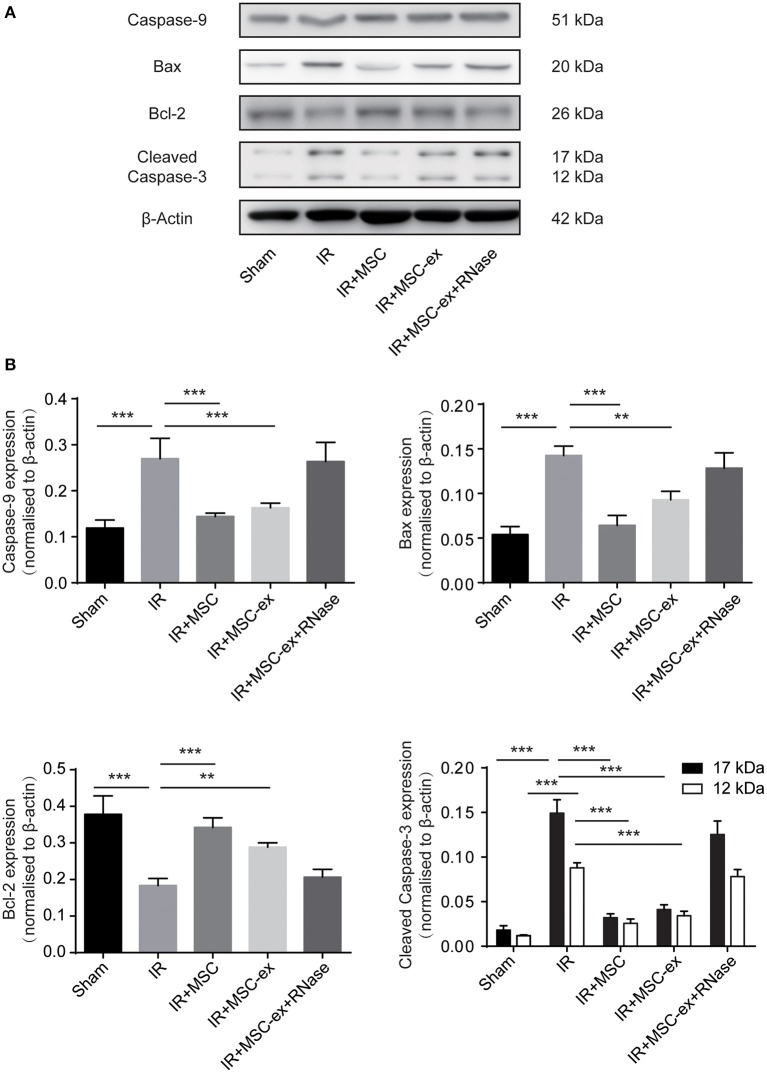
MSC and exosomes from MSC reduced apoptosis by inhibiting expression levels of caspase-9, cleaved caspase-3, and Bax, and increasing expression level of Bcl-2. **(A)** Representative images show the protein levels of caspase-9, Bax, Bcl-2, and cleaved caspase-3. **(B)** The expression levels of caspase-9, Bax, Bcl-2, and cleaved caspase-3 were normalized to β-actin levels within the same sample. ^**^P < 0.01, ^***^P < 0.001, *n* = 6.

As demonstrated earlier, cell apoptosis increased after IRI. Additionally, treatment with MSC and MSC-ex significantly decreased the number of apoptotic cells (Figures 1A,B). In the subsequent experiment, we examined the expression levels of apoptosis-related proteins, such as caspase-9, Bax, and cleaved caspase-3 to further verify the anti-apoptotic effects of MSC and exosomes from MSC. Results showed that both of them notably inhibited the expression levels of caspase-9 in kidneys after IRI, as well as cleaved caspase-3, compared with the IRI group. However, MSC and exosomes from MSC downregulated the expression levels of the pro-apoptotic proteins (caspase-9, cleaved caspase-3, and Bax), while upregulated the expression level of the anti-apoptotic protein, Bcl-2, indicating that both of them inhibited IR-induced apoptosis. Furthermore, the expression levels of caspase-9, Bax, and cleaved caspase-3 were slightly higher, whereas the expression level of Bcl-2 was lower in the MSC-ex group compared with the MSC group. The expression levels of these proteins were similar in the IR + MSC-ex + RNAase group and IRI group (Figure 4).

## Discussion

A previous study demonstrated that MSCs can repair IR-induced AKI (18, 19). MSCs have shown promising effects in experimental models of AKI and CKD, and have been used in clinical practice for more than one decade. The regenerative effects of MSCs do not rely on their ability to differentiate and replace damaged tissue, while those effects are primarily mediated by the release of paracrine factors (20). Therefore, we hypothesized that MSCs affect AKI rats via paracrine network.

In the immune system, exosomes have been demonstrated to play a pivotal role in mediating both adaptive and innate immune responses, thereby participating in antigen presentation associated with MHC-II and MHC-I molecules (9, 21). It has been suggested that aggregation of MSCs, which could differentiate into renal tubular epithelial cells, is directly involved in the repair and reconstruction of renal tubules mediated by chemotaxis, with homing ability in injured kidneys (22, 23). In the present studys, we detected protective effect of MSCs and MSC-ex on renal IRI, respectively. The results confirmed that MSCs and MSC-ex could alleviate tissue injuries and apoptosis after IRI. We found that treatment with MSC and MSC-ex significantly attenuated pathological damage to the kidney. To investigate the specific mechanism, we examined the expression levels of associated pro-inflammatory and transcription factors. The mRNA levels of TNF-α, NF-κB, IFN-γ, and IL-6 were noticeably after undergoing MSC and MSC-ex, suggesting that MSCs and exosomes could inhibit the inflammatory response in renal IRI (Figure 2). TNF-α has been deemed to play a substantial factor in the inflammatory cascade and systemic inflammatory response (24), resulting in aggravation of renal IRI.

NF-κB can be activated in kidney cells during IRI, and is highly involved in immune responses (17, 25–27). It is activated through phosphorylation of IκB, followed by proteasome-mediated degradation. Accordingly, we tested the expression levels of NF-κB, IκB, p-NF-κB, and p-IκB (15, 17, 25, 26). In the current research, it was unveiled that the expression levels of p-NF-κB and p-IκB were decreased after undergoing MSC and MSC-ex, while the expression level of IκB was increased. However, in the MSC-ex + RNAase group, the expression levels of NF -κB, IκB, p- NF-κb, and p- IκB were similar to those in the IR group. These results suggest that both MSC and MSC-ex can inhibit NF-κB activation after IRI, and RNA hydrolase can eliminate the effects of MSC-ex.

Apoptosis is a marker of severe tissue injury in IRI. We, in the present study, observed significant reduction of apoptotic cells in the IR + MSC-ex group compared with the IR group, providing further evidence for protective effects of MSC-ex on renal IRI. Cleaved caspase-3 plays a significant role in the downstream signaling pathway. During the renal IRI, Bax/Bcl-2 ratio could be upregulated to activate mitochondria-mediated apoptosis and release intracellular cytochrome C (28–30). In the present study, we found that MSCs and exosomes inhibited the expression levels of cleaved caspase-3, caspase-9 and Bax, and upregulated expression level of Bcl-2, confirming the effects of MSCs and exosomes on mitochondria-mediated apoptosis (Figure 4). However, we also observed that exosomes alone were not as effective as MSCs, suggesting that exosomes may be the main effector of MSCs on anti-inflammatory function. MSCs may be able to exert protective effect through other mechanisms, such as releasing cytokines.

Taken together, the results of this study demonstrate that exosomes originating from MSCs have protective effects on IRI via inhibition of apoptosis and inflammatory response, in which NF-κB signaling pathway may play a key role.

## Data Availability Statement

The datasets analyzed in this manuscript are not publicly available. Requests to access the datasets should be directed to ljlmjw@163.com.

## Ethics Statement

All animal procedures were performed in accordance with the guidelines of the bioethics, and was approved by the Bioethics Committee of Zhongshan Hospital, Fudan University, Shanghai.

## Author Contributions

LL, RW, and YJ carried out the molecular biology studies and the immunoassays, analyzed the data, and drafted the manuscript. RR performed the cell experiments. TZ and MX designed and supervised the study, revised the manuscript, and gave final approval for publication.

### Conflict of Interest

The authors declare that the research was conducted in the absence of any commercial or financial relationships that could be construed as a potential conflict of interest.

## Supplementary Material

Supplementary Image 1 The identification of isolated exosomes by TEM.

Supplementary Image 2 The exosomal protein CD63 was verified by flow.

## References

[1] Eltzschig HK Eckle T. Ischemia and reperfusion–from mechanism to translation. Nat Med. (2011) 17:1391–401. 10.1038/nm.2507 22064429 PMC3886192

[2] Kim SP Thompson RH. Kidney function after partial nephrectomy: current thinking. Curr Opin Urol. (2013) 23:105–11. 10.1097/MOU.0b013e32835d8ec1 23321635

[3] Wood KJ Goto R. Mechanisms of rejection: current perspectives. Transplantation. (2012) 93:1–10. 10.1097/TP.0b013e31823cab44 22138818

[4] van den Akker EK Manintveld OC Hesselink DA de Bruin RW Ijzermans JN Dor FJ. Protection against renal ischemia-reperfusion injury by ischemic postconditioning. Transplantation. (2013) 95:1299–305. 10.1097/TP.0b013e318281b934 23519023

[5] Saat TC van den Akker EK IJzermans JN Dor FJ de Bruin RW. Improving the outcome of kidney transplantation by ameliorating renal ischemia reperfusion injury: lost in translation? J Transl Med. (2016) 14:20. 10.1186/s12967-016-0767-2 26791565 PMC4721068

[6] Uccelli A Moretta L Pistoia V. Mesenchymal stem cells in health and disease. Nat Rev Immunol. (2008) 8:726–36. 10.1038/nri2395 19172693

[7] Furuichi K Shintani H Sakai Y Ochiya T Matsushima K Kaneko S. Effects of adipose-derived mesenchymal cells on ischemia-reperfusion injury in kidney. Clin Exp Nephrol. (2012) 16:679–89. 10.1007/s10157-012-0614-6 22398959

[8] Katsha AM Ohkouchi S Xin H Kanehira M Sun R Nukiwa T. Paracrine factors of multipotent stromal cells ameliorate lung injury in an elastase-induced emphysema model. Mol Ther. (2011) 19:196–203. 10.1038/mt.2010.192 20842104 PMC3017437

[9] Lange C Togel F Ittrich H Clayton F Nolte-Ernsting C Zander AR. Administered mesenchymal stem cells enhance recovery from ischemia/reperfusion-induced acute renal failure in rats. Kidney Int. (2005) 68:1613–7. 10.1111/j.1523-1755.2005.00573.x 16164638

[10] Deregibus MC Cantaluppi V Calogero R Lo Iacono M Tetta C Biancone L. Endothelial progenitor cell derived microvesicles activate an angiogenic program in endothelial cells by a horizontal transfer of mRNA. Blood. (2007) 110:2440–8. 10.1182/blood-2007-03-078709 17536014

[11] Li T Yan Y Wang B Qian H Zhang X Shen L. Exosomes derived from human umbilical cord mesenchymal stem cells alleviate liver fibrosis. Stem Cells Dev. (2013) 22:845–54. 10.1089/scd.2012.0395 23002959 PMC3585469

[12] Liao Z Luo R Li G Song Y Zhan S Zhao K. Exosomes from mesenchymal stem cells modulate endoplasmic reticulum stress to protect against nucleus pulposus cell death and ameliorate intervertebral disc degeneration *in vivo*. Theranostics. (2019) 9:4084–100. 10.7150/thno.33638 31281533 PMC6592170

[13] He J Wang Y Sun S Yu M Wang C Pei X. Bone marrow stem cells-derived microvesicles protect against renal injury in the mouse remnant kidney model. Nephrology. (2012) 17:493–500. 10.1111/j.1440-1797.2012.01589.x 22369283

[14] Zhou Y Xu H Xu W Wang B Wu H Tao Y. Exosomes released by human umbilical cord mesenchymal stem cells protect against cisplatin-induced renal oxidative stress and apoptosis *in vivo* and *in vitro*. Stem Cell Res Ther. (2013) 4:34. 10.1186/scrt194 23618405 PMC3707035

[15] Brines M Cerami A. The receptor that tames the innate immune response. Mol Med. (2012) 18:486–96. 10.2119/molmed.2011.00414 22183892 PMC3356428

[16] Lin M Li L Li L Pokhrel G Qi G Rong R. The protective effect of baicalin against renal ischemia-reperfusion injury through inhibition of inflammation and apoptosis. BMC Complement Altern Med. (2014) 14:19. 10.1186/1472-6882-14-19 24417870 PMC3893527

[17] Mitchell S Vargas J Hoffmann A. Signaling via the NFkappaB system. Wiley Interdiscip Rev Syst Biol Med. (2016) 8:227–41. 10.1002/wsbm.1331 26990581 PMC8363188

[18] Cao H Qian H Xu W Zhu W Zhang X Chen Y. Mesenchymal stem cells derived from human umbilical cord ameliorate ischemia/reperfusion-induced acute renal failure in rats. Biotechnol Lett. (2010) 32:725–32. 10.1007/s10529-010-0207-y 20131083

[19] Alzahrani FA. Melatonin improves therapeutic potential of mesenchymal stem cells-derived exosomes against renal ischemia-reperfusion injury in rats. Am J Transl Res. (2019) 11:2887–907. 31217862 PMC6556638

[20] Chen YT Sun CK Lin YC Chang LT Chen YL Tsai TH. Adipose-derived mesenchymal stem cell protects kidneys against ischemia-reperfusion injury through suppressing oxidative stress and inflammatory reaction. J Transl Med. (2011) 9:51. 10.1186/1479-5876-9-51 21545725 PMC3112438

[21] van Kooten C Rabelink TJ de Fijter JW Reinders MEJ. Mesenchymal stromal cells in clinical kidney transplantation. Curr Opin Organ Transplant. (2016) 21:550–8. 10.1097/MOT.0000000000000364 27755168

[22] Kale S Karihaloo A Clark PR Kashgarian M Krause DS Cantley LG. Bone marrow stem cells contribute to repair of the ischemically injured renal tubule. J Clin Invest. (2003) 112:42–9. 10.1172/JCI17856 12824456 PMC162291

[23] Li N Li XR Yuan JQ. Effects of bone-marrow mesenchymal stem cells transplanted into vitreous cavity of rat injured by ischemia/reperfusion. Graefes Arch Clin Exp Ophthalmol. (2009) 247:503–14. 10.1007/s00417-008-1009-y 19084985

[24] Furuichi K Kokubo S Hara A Imamura R Wang Q Kitajima S. Fas ligand has a greater impact than TNF-alpha on apoptosis and inflammation in ischemic acute kidney injury. Nephron Extra. (2012) 2:27–38. 10.1159/000335533 22479266 PMC3318938

[25] Sanz AB Sanchez-Nino MD Ramos AM Moreno JA Santamaria B Ruiz-Ortega M. NF-kappaB in renal inflammation. J Am Soc Nephrol. (2010) 21:1254–62. 10.1681/ASN.2010020218 20651166

[26] Diamant G Dikstein R. Transcriptional control by NF-κB: elongation in focus. Biochim Biophys Acta. (2013) 1829:937–45. 10.1016/j.bbagrm.2013.04.007 23624258

[27] Prakoura N Kavvadas P Kormann R Dussaule JC Chadjichristos CE Chatziantoniou C. NFkappaB-induced periostin activates integrin-beta3 signaling to promote renal injury in GN. J Am Soc Nephrol. (2016) 28:1475–90. 10.1681/ASN.2016070709 27920156 PMC5407726

[28] Yang B Jain S Ashra SY Furness PN Nicholson ML. Apoptosis and caspase-3 in long-term renal ischemia/reperfusion injury in rats and divergent effects of immunosuppressants. Transplantation. (2006) 81:1442–50. 10.1097/01.tp.0000209412.77312.69 16732183

[29] Brooks C Wei Q Cho SG Dong Z. Regulation of mitochondrial dynamics in acute kidney injury in cell culture and rodent models. J Clin Invest. (2009) 119:1275–85. 10.1172/JCI37829 19349686 PMC2673870

[30] Wei Q Dong G Chen JK Ramesh G Dong Z. Bax and Bak have critical roles in ischemic acute kidney injury in global and proximal tubule-specific knockout mouse models. Kidney Int. (2013) 84:138–48. 10.1038/ki.2013.68 23466994 PMC3686831

