# Supplementary figures and images for: Exosomes Derived From Mesenchymal Stem Cells Ameliorate Renal Ischemic-Reperfusion Injury Through Inhibiting Inflammation and Cell Apoptosis
Source: Front Med (Lausanne). 2019 Nov 19;6:269. doi: 10.3389/fmed.2019.00269 (PMC6907421; doi:10.3389/fmed.2019.00269)

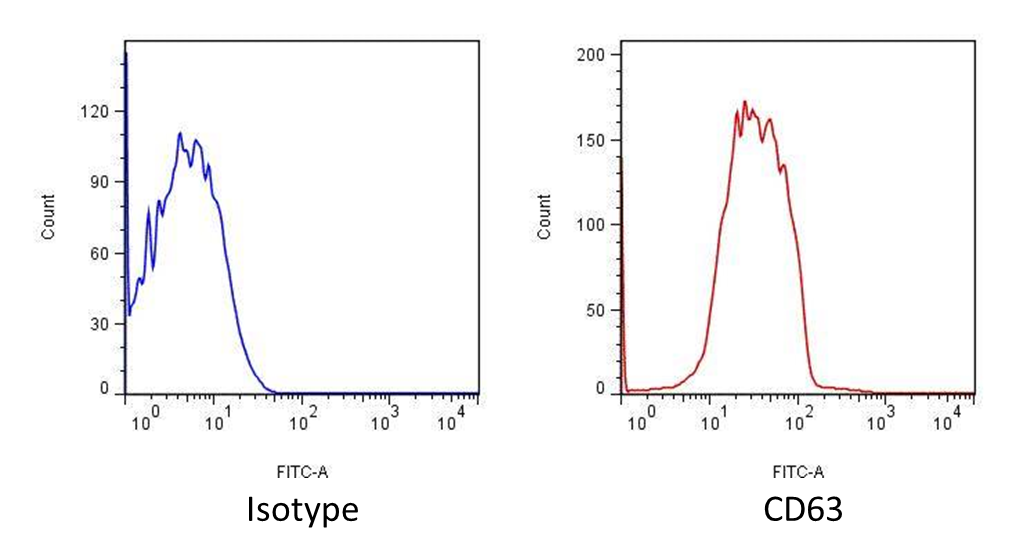

Supplement: Supplementary Image 2 — The exosomal protein CD63 was verified by flow. [file Image_2.PNG]
